# The Influence of Gender and Education on Awareness About Sexually Transmitted Diseases Among King Saud University and Imam Mohammed Ibn Saud Islamic University Students in Riyadh, Saudi Arabia

**DOI:** 10.7759/cureus.54998

**Published:** 2024-02-26

**Authors:** Mohammed ALDosari, Thiab Abuzied, Nawaf Alhussaini, Mohammed Althobaiti, Yazeed Tumihi, Talal Alhumaid, Abdullah AlRumayan

**Affiliations:** 1 Family Medicine, King Saud Bin Abdulaziz University for Health Sciences College of Medicine, Riyadh, SAU; 2 Family Medicine and Primary Care, King Abdulaziz Medical City, Riyadh, SAU; 3 Family and Community Medicine, King Saud Bin Abdulaziz University for Health Sciences College of Medicine, Riyadh, SAU

**Keywords:** hiv diseases, educational level, university students, riyadh - saudi arabia, sexually transmitted diseases (stds)

## Abstract

Background: Sexually transmitted diseases (STDs) are a global issue facing the world. In a conservative community like Saudi Arabia, discussing such matters is considered taboo, and this might impact the awareness of STDs. Therefore, this study aims to determine if gender, level of education, and university affiliation influence the level of awareness of STDs.

Methods: This research is a cross-sectional study that involves 389 students who are studying at King Saud University (KSU) and Imam Mohammad Ibn Saud Islamic University (IMAMU). Twenty-six questions were gathered from a previous study and implemented into the questionnaire and were validated after.

Results: Overall, awareness about STDs was poor. Females scored higher than male participants (7.9 ± 3.3 vs. 7.1 ± 3.2, p < 0.017) respectively. Interestingly, undergraduates scored higher than postgraduates (4.7 ± 3.7 vs. 3.7 ± 3.3, p < 0.029). For the university, KSU students scored higher than their counterparts did at IMAMU (10.4 ± 5.7 vs. 8.9 ± 5.5, p < 0.01).

Conclusion: Female participants have shown a higher level of STD awareness than males, but the overall awareness is still extremely low. However, KSU scored higher than IMAMU As a result, appropriate education and promotion efforts about STDs must be implemented based on gender and education level.

## Introduction

“Sexually transmitted diseases” (STDs), also known as sexually transmitted infections (STIs), is a vast term that encompasses more than 30 miscellaneous clinical syndromes and infections that can invade the body via sexual contact [1,2]. Several species of bacteria, viruses, and parasites can create an environment ripe for developing STDs, such as chlamydia infection and gonorrhea [2]. In compliance with the recent statistics from the World Health Organization (WHO), STDs pose a significant public health challenge in both developing and developed countries since they can be correlated with serious complications and thereby poor quality of life [2]. In 2016, the global total of new curable STDs was estimated at approximately 376.4 million, half of which are gonorrhea and chlamydia, in the age group between 15 and 49 years [3]. In other words, more than one million individuals are diagnosed with curable STDs each day, so STDs are one of the top five reasons for seeking healthcare in developing countries [3].

In Saudi Arabia, over eight years from January 2005 to December 2012, 68,886 cases were reported to the Ministry of Health (MOH), with an annual incidence of 92.1/100,000 [4]. Furthermore, a comparison of STDs in 1995-1999 and 2005-2012 revealed the escalating epidemic trend of STD cases in Saudi Arabia, where nongonococcal urethritis increased from 37.3% to 51.7%, human immunodeficiency virus (HIV) from 7.5% to 14.2%, human papillomavirus (HPV) from 3.5% to 5.8%, and genital herpes from 0.6% to 2.1% [4,5]. The contribution of Saudi citizens to HIV cases was raised by 14.6% (from 28.9% to 43.5%) in the preceding decade [4,5]. According to the recent announcement from the WHO, awareness of STDs serves as the first line of defense to curtail these alarming statistics about the rate of STD incidence and prevalence in the long run [2,6].

Despite the value of awareness of the population, previous studies have shown overall low levels of awareness of STDs among the Middle Eastern population [7-9]. Moreover, conservative societies are more likely to lack awareness of STDs [9-11]. Due to both cultural and religious considerations, Saudi Arabian society is not an exception where STDs remain a sensitive topic, and any trigger to discuss STDs is taboo, thereby indicating poor knowledge [12]. In addition, the risk seems to exacerbate since STD patients can be asymptomatic and remain undiagnosed [1]. All of that precludes patients from seeking help with early diagnosis and effective treatment of their partners [6,13]. Hence, delayed treatment leads to severe complications such as the acquisition of HIV and infertility, genital malignancies, pelvic inflammatory disease, and systemic infection. In addition, psychological consequences like anxiety, depression, and stigma. Thus, resulting in a high prevalence of HIV cases and associated complications. Nevertheless, Saudi Arabia encounters a definite shortage of appropriate research being conducted on STDs, in the sense that research has left much to be desired, especially when it comes to the knowledge level, attitudes, risky behaviors, and preventive practices of these diseases [12]. The present study aimed to identify the level of awareness of STDs among Saudi KSU and IMAMU students in Riyadh, with special attention to the assessment of the influence of gender and education on the level of awareness.

## Materials and methods

Study design, settings, and participants

 We implemented a descriptive cross-sectional study through a survey conducted in Saudi Arabia, Riyadh city. A self-administered online questionnaire that categorizes participants’ responses through the Likert Scale was conducted using Google Forms. The study population consisted of 389 students from King Saud University (KSU) and Imam Mohammed Ibn Saud Islamic University (IMAMU) who are between 18 and 24 years old regardless of gender. The recruitment of participants and data collection were carried out between November 2021 and March 2022. Those who are graduate and undergraduate students were included as for the exclusion of medical students. A non-probability convenience sample technique was used. All students who were willing to participate in the study and available during data collection were included in the study.

Questionnaires and data collection process

Ethical approval was obtained from the Institutional Review Board vide Number IRBC/1307/21 dated April 7, 2021 and informed written consent was taken from all study participants. A 26-item questionnaire consisting of four sections was used for data collection. In addition to the demographic data, eight questions for general knowledge, four questions for mode of transmission and causes, six questions for manifestation, and eight questions for management were employed to evaluate the awareness of sexually transmitted diseases among KSU and IMAMU students. Respondents were asked to answer in limited Likert Scale formats. The primary version of the questionnaire was developed in a study that was conducted to measure the knowledge of STDs [14]. We contacted three consultants in infectious diseases at NGHA for validation. The scoring was individualized based on each section as follows, Mode of Transmission has four points for each item, Management has three points for each item, General Knowledge has two points for each item, and Manifestation has one point for each item. Items were assessed with five response options (“Agree,” “Strongly Agree,” “Neutral,” “Disagree,” “Strongly Disagree”) based on the correctness of each item, the scores were considered the total point of the item whether it was “Agree, Strongly Agree” or “Disagree, Strongly Disagree.” However, wrong choices and “Neutral” options were assessed as “Zero Point’’ based on each item in each section. Respondents who got more than 50% of the total score were considered “Aware”. On the other hand, respondents who got less than fifty percent of the total score were considered as “unaware.”

Statistical analysis

Data processing and analysis were carried out using the JMP software. The analysis included descriptive statistics such as percentages and frequencies, means, and standard deviations, which were performed in order to assess the sample characteristics. One-way ANOVA test was used to compare the difference between the demographic factors and level of awareness. A Pearson correlation test was used for the correlation between each section and the total score. A p-value of 0.05 was considered a significance level for all the statistical tests.

## Results

About 388 participants were involved in this study. Of those participants (36.6%) were male and (63.4%) were female. Most of the participants (65.7%) were under 23 years old compared to 28.1% who were between 23 and 30 years old, and only a few (6.2%) were above 30 years. Almost all the participants (89%) were single, 9.8% were married, and 1.5% were separated. Most of the participants were undergraduate (80%) and only 20% were postgraduate as seen in Table 1.

**Table 1:** Sociodemographic characteristics of participants

Category	Subcategory	N (%)
Age	Under 23 years old	255 (65.7%)
	Between 23 and 30 years old	109 (28.1%)
	Above 30 years old	24 (6.2%)
Gender	Females	246 (63.4%)
	Males	142 (36.6%)
Level of education	Undergraduate	310 ( 79.9%)
	Postgraduate	78 (20.1%)
Marital status	Single	344 (88.7%)
	Married	38 (9.8%)
	separated	6 (1.5%)
Financial income	Less than your need	112 (28.9%)
	Meet your needs	227 (58.5%)
	More than your need	49 (12.6%)
University	King Saud university	188 (48.5%)
	Imam Muhammad ibn Saud Islamic university	200 (51.5%)

The management section has the highest effect on total score (Table 2).

**Table 2:** Total score correlation of general knowledge, mode of transmission, manifestation, and management of sexually transmitted diseases

Section	General knowledge (max=16)	Mode of transmission and causes (max=16)	Manifestation (max=6)	Management (24=max)
General knowledge (max=16)	1			
Mode of transmission and causes (max=16)	**299	1		
Manifestation (max=6)	**381	**357	1	
Management (24=max)	**514	**253	**466	1
Total score(max=62)	**750	**630	**625	**825

The general knowledge section scored the highest level of knowledge among all sections (48%, n = 185) (Table 3). Expectedly, the largest percentage of respondents identified that HIV weakens the immune system, and that HIV causes AIDS (85%, n = 330; 76.5%, n= 297), respectively (Table 4).

**Table 3:** Descriptive statistics knowledge, transmission, manifestation, and management sexually transmitted diseases

Section	Mean	SD	N (%)
General knowledge (max=16)	7.6	3.3	185 (48%)
Mode of transmission and causes (max=16)	4.5	3.7	109 (28%)
Manifestation (max=6)	1.6	1.3	101 (26%)
Management (24=max)	9.7	5.6	155 (40%)
Total score (max=62)	23.3	10.4	147 (38%)

**Table 4:** Level of awareness about sexually transmitted diseases

Questions	Strongly disagree	Disagree	Neutral	Agree	Strongly agree	Overall
	N (%)	N (%)	N (%)	N ( %)	N (%)	Agreement N (%)
The same virus causes all of the Sexually Transmitted Diseases.	74 (19.1%)	135 (34.8%)	138 (35.6%)	24 (6.2%)	17 (4.4%)	41 (10.6%)
Genital Herpes can lead to death in adults.	21 (5.4%)	53 (13.7%)	239 (61.6%)	55 (14.2%)	20 (5.2%)	75 (19.4%)
Sexually Transmitted Diseases can lead to health problems that are usually more serious for men than women.	32 (8.2%)	98 (25.3%)	182 (46.9%)	46 (11.9%)	30 (7.7%)	76 (19.6%)
Women are more likely to get HIV during vaginal sex than men.	18 (4.6%)	34 (8.8%)	183 (47.2%)	85 (21.9%)	68 (17.5%)	153 (39.4%)
A pap smear may tell if a woman has Human Papillomavirus (HPV).	0 (0.0%)	8 (2.1%)	191 (49.2%)	99 (25.5%)	90 (23.2%)	189 (48.7%)
Untreated Sexually Transmitted Diseases can develop into other Sexually Transmitted Diseases.	9 (2.3%)	27 (7.0%)	159 (41.0%)	116 (29.9%)	77 (19.8%)	193 (49.7%)
HIV is the virus that causes AIDS.	8 (2.1%)	18 (4.6%)	65 (16.8%)	132 (34.0%)	165 (42.5%)	297 (76.5%)
HIV lowers the ability of a person’s body to fight off diseases.	1 (0.3%)	6 (1.5%)	51 (13.1%)	115 (29.6%)	215 (55.4%)	330 (85.0%)
The cause of Genital Herpes is unknown.	33 (8.5%)	92 (23.7%)	224 (57.7%)	22 (5.7%)	17 (4.4%)	39 (10.1%)
A woman who has Genital Herpes can pass the infection to her baby during childbirth.	13 (3.4%)	71 (18.3%)	211 (54.4%)	62 (16.0%)	31 (8.0%)	93 (24.0%)
Chlamydia can be transmitted to another person during oral sex.	6 (1.5%)	17 (4.4%)	247 (63.7%)	70 (18.0%)	48 (12.4%)	118 (30.4%)
It is easier to get HIV if a person has another Sexually Transmitted Disease.	5 (1.3%)	36 (9.3%)	151 (38.9%)	123 (31.7%)	73 (18.8%)	196 (50.5%)
Chlamydia can lead to infertility in women.	7 (1.8%)	21 (5.4%)	273 (70.4%)	52 (13.4%)	35 (9.0%)	87 (22.4%)
Soon after infection with HIV a person develops open sores on his or her genitals (penis or vagina).	9 (2.3%)	21 (5.4%)	234 (60.3%)	89 (22.9%)	35 (9.0%)	124 (31.9%)
A woman can tell that she has Chlamydia if she has a bad smelling odor from her vagina.	7 (1.8%)	21 (5.4%)	223 (57.5%)	84 (21.6%)	53 (13.7%)	137 (35.3%)
The early symptoms of HIV can be the same as the flu.	8 (2.1%)	35 (9.0%)	205 (52.8%)	90 (23.2%)	50 (12.9%)	140 (36.1%)
It can take several years after being infected with HIV for a person to develop AIDS.	10 (2.6%)	34 (8.8%)	164 (42.3%)	100 (25.8%)	80 (20.6%)	180 (46.4%)
The symptoms of Sexually Transmitted Diseases can be the same as the symptoms of other diseases.	5 (1.3%)	22 (5.7%)	148 (38.1%)	155 (39.9%)	58 (14.9%)	213 (54.8%)
The birth control patch can protect a woman from getting a Sexually Transmitted Disease.	77 (19.8%)	127 (32.7%)	153 (39.4%)	13 (3.4%)	18 (4.6%)	31 (8.0%)
A person should stop taking medications for a Sexually Transmitted Disease when the symptoms disappear.	87 (22.4%)	94 (24.2%)	174 (44.8%)	20 (5.2%)	13 (3.4%)	33 (8.6%)
There is a vaccine available that can reduce the health problems caused by AIDS.	38 (9.8%)	49 (12.6%)	202 (52.1%)	66 (17.0%)	33 (8.5%)	99 (25.5%)
Using a dental dam during oral sex can protect a person from getting a Sexually Transmitted Disease.	28 (7.2%)	61 (15.7%)	191 (49.2%)	68 (17.5%)	40 (10.3%)	108 (27.8%)
A woman who has HIV can be cured if treated soon after she gets it.	27 (7.0%)	50 (12.9%)	193 (49.7%)	87 (22.4%)	31 (8.0%)	118 (30.4%)
Some medications are available to treat HIV.	32 (8.2%)	48 (12.4%)	164 (42.3%)	106 (27.3%)	38 (9.8%)	144 (37.1%)
There are medications that can reduce the health problems caused by Sexually Transmitted Diseases.	11 (2.8%)	12 (3.1%)	143 (36.9%)	153 (39.4%)	69 (17.8%)	222 (57.2%)
Using a female condom during vaginal sex can help a woman avoid HIV infection.	8 (2.1%)	37 (9.5%)	118 (30.4%)	131 (33.8%)	94 (24.2%)	225 (58.0%)

In management section, yielded 40% (n = 154) knowledge (Table 3). Exceedingly more than 50% (58%, n = 225), most of the participants were aware that vaginal sex can prevent HIV infection (Table 4). Majority of the participants knew that there are medications that can reduce the effects of STDs. Next is mode of transmission and causes, in which the level of knowledge was (28%, n = 107) (Table 3). In this section, participants were asked about how STDs are transmitted. Particularly, there were questions about how genital herpes and chlamydia are transmitted. Minority of the participants were aware how genital herpes (24%, n = 93) and chlamydia (30%, n = 118) are transmitted (Table 4). Concerning manifestation, the overall level of knowledge in this section was 26% (n = 100) (Table 3). Results showed that more than 50% (54.8, n = 213) of participants knew the manifestation of STDs could resemble a symptom of another disease (Table 4). It was noticeable that only 46.4% (n = 180) of participants knew that HIV causes AIDS, and 36.1% (n = 140) were aware that the early symptoms of HIV can be the same as the flu (Table 4). Female participants showed a higher mean and standard deviation in general knowledge compared to males (7.9 ± 3.3 vs. 7.1 ± 3.2, p < 0.017) (Table 5).

**Table 5:** Relationship between demographic characteristics of participants and their knowledge about sexually transmitted diseases

Subcategory		General Knowledge (max=16)		Mode of Transmission and Causes (max=16)		Manifestation(max=6)		Management(max=24)		Total score (max=62)	
		Mean	SD	Mean	SD	Mean	SD	Mean	SD	Mean	SD
Age	<23 years old	7.5	3.3	4.6	3.5	1.5	1.2	9.3	5.4	23	10.2
	from 23 and 30 years old	7.5	3.3	4.1	3.9	1.6	1.4	10.2	6.1	23.4	10.9
	>30 years old	8.9	3.4	5	4.1	1.7	1.5	10.5	6	26.1	10.5
	P-value	0.13		0.432		0.612		0.318		0.371	
Gender	Male	7.1	3.2	4.1	3.7	1.4	1.2	9.9	5.8	22.5	10.7
	Female	7.9	3.3	4.7	3.6	1.6	1.3	9.5	5.6	23.8	10.2
	P-value	0.017		0.087		0.106		0.527		0.221	
Marital status	Single	3	3.3	4.5	3.6	1.5	1.3	9.5	5.7	23.1	10.6
	Married	8	3.2	4.6	3.9	1.4	1.3	10.7	5.4	24.7	8.9
	Separated	10.3	2.3	4	5.1	2.5	1.8	10.5	5.6	27.3	7.4
	P-value	0.085		0.924		0.154		0.466		0.422	
Financial income	Less than your need	7.6	3.1	4.4	3.6	1.5	1.3	9.3	5.5	22.8	9.3
	Meet your needs	7.6	3.3	4.4	3.6	1.6	1.3	9.5	5.6	23.1	10.5
	More than your need	7.8	3.7	5.1	4.1	1.6	1.4	10.9	6	25.3	12
	P-value	0.864		0.524		0.954		0.242		0.331	
Level of Education	Undergraduate	7.5	3.3	4.7	3.7	1.5	1.3	9.5	5.6	23.2	10.4
	Postgraduate	8.1	3.4	3.7	3.3	1.6	1.4	10.4	5.6	23.8	10.4
	P-value	0.113		0.029		0.684		0.174		0.605	
University	King Saud University	7.8	3.3	4.4	3.6	1.5	1.3	10.4	5.7	24.2	10.4
	IMAMU	7.4	3.3	4.6	3.7	1.6	1.3	8.9	5.5	22.4	10.4
	P-value	0.181		0.762		0.803		0.01		0.094	

Furthermore, those who were in KSU had a higher mean and standard deviation in management compared to Imam Muhammad ibn Saud Islamic University (10.4 ± 5.7 vs. 8.9 ± 5.5, p < 0.01). unexpectedly undergraduate students showed a higher mean and standard deviation in Mode of Transmission and Causes compared to postgraduate students (4.7 ± 3.7 vs. 3.7 ± 3.3, p < 0.029).

## Discussion

There is a lack of participant awareness regarding the STD manifestation, mode of transmission and causes, management, and general knowledge respectively. The conservative cultural nature of Saudi society stands behind this lack of awareness of STDs since Saudi individuals believe discussing such issues is the path to moral decay and licentiousness [12,14]. Moreover, religion could serve as a double-edged sword for Saudi teenagers [15]. For example, religion obligates abstinence before marriage and male circumcision which have been strongly associated with lower HIV and other STDs prevalence, whereas it may prevent them from discussing STDs freely with family members, teachers, and even healthcare providers [16,17]. As a consequence, 71.7% of surveyed Saudis in a previous study considered the Internet as the main source of knowledge about STDs [8]. In addition, several studies have affirmed that misinformation and misconceptions about STDs among Saudi teenagers are widespread [14,18]. Therefore, it is wise to emphasize that religious rules and precepts never oppose talking about STDs, and the message of preventive education about STDs be introduced in the context of Islamic religion and Saudi culture.

 We confirmed gender as a predictor of STD awareness. Female participants have a higher overall awareness of STDs compared to males, which is consistent with several previous global and local studies [12,19,20]. Nevertheless, the level of awareness of STDs in relation to management and prevention among female participants is far from adequate compared to males. These gender differences are possibly due to the fact that women are at higher risk of the most serious sequelae of STDs, such as cancer, than men [21,22]. On the other hand, men have an equal probability of acquisition and transmission of STDs to women [21,22]. As a consequence, this suggests timely STD intervention and treatment among men will be less likely when STD symptoms arise, and thereby the high probability of acquisition of HIV [2,6]. Hence, in order to enhance awareness of STDs, education and promotion must be ducted with considerations of gender.

Postgraduate students have shown a higher level of awareness of STDs compared to their counterparts at the undergraduate level. This might be explained by that postgraduate students are more likely to be exposed to basic STDs-related information since they are more immersed and experienced in life. On the other hand, despite lacking ample knowledge of STDs, undergraduate students are more exposed to pornographic materials, thus resulting in widespread risky behaviors [23]. Unlike postgraduate students, who expected to show higher awareness of STDs regarding mode of transmission and causes, undergraduate students showed higher awareness compared to their counterparts at the postgraduate level. This finding presses the need for further investigation to identify better the actual reason behind such an improvement in the level of awareness shown by undergraduate students.

University type is also the predictor of knowledge level, so KSU students were more likely to have good knowledge than IMAMU students. This is possibly due to disparities in the nature and the quality and quantity of educational campaigns about STDs in both two universities. However, Saudi universities have not yet applied an evidence-based health education program about STDs at any education level, whereas sex education has already become compulsory in many European countries. Moreover, many longitudinal surveys in Europe reaffirm that continuous educational campaigns have significantly improved awareness of STDs among students, but there were only some campaigns targeted to enhance awareness about sexual and behavioral abuse among young students in Saudi Arabia [24]. As a consequence, Saudi universities must integrate evidence-based health educational programs into undergraduate academia. That is accomplished either via health-driven curricula, health-related electives, or mandatory courses for graduation. These efforts will increase awareness of STDs and shorten the risk of acquisition of HIV from STD cases and the treatment-seeking time of STD patients. Hence, they will improve their quality of life, reduce the socioeconomic burden of STDs, and positively influence public health policies.

Limitations

The study has some limitations. Studies targeted to determine the level of awareness of STDs usually include investigations of perceptions and attitudes toward STDs, but it was difficult to involve attitudes and practices towards STDs in this study due to social desirability bias and fear of judgment. Therefore, since it may also result in harassment for the participants, any questions investigating the attitude and practice toward STDs have already been excluded. Furthermore, participants involved in this study were only university students, so the generalizability of findings from this study among the youth of the Riyadh population might be limited. Future research must include school or university dropouts, as well as young adults who are not enrolled in university, because they are more likely to engage in risky sexual behaviors. However, this study is the first of its kind to determine the level of awareness of STDs among Saudi KSU and IMAMU students in Riyadh, Saudi Arabia.

## Conclusions

Awareness of STDs is essential for individuals to be able to adopt preventive actions and healthy behaviors. Female participants have shown a higher level of awareness than males, but the overall awareness is still extremely low. In order to enhance awareness of STDs among university students, appropriate education and promotion efforts must be implemented based on gender and education level. Active efforts by the government, educational institutions, and medical institutions are necessary to improve awareness of STDs.
